# Hypertension and Postoperative Pain: A Prospective Observational Study

**DOI:** 10.1155/2019/8946195

**Published:** 2019-01-09

**Authors:** Han-Liang Chiang, Yu-Chi Huang, Huey-Shyan Lin, Min-Ho Chan, Yuan-Yi Chia

**Affiliations:** ^1^Department of Anesthesiology, Kaohsiung Veterans General Hospital, Kaohsiung 813, Taiwan; ^2^School of Medicine, National Defense Medicine Center, Taipei 114, Taiwan; ^3^School of Nursing, Fooyin University, Kaohsiung 831, Taiwan; ^4^School of Medicine, National Yang-Ming University, Taipei 112, Taiwan

## Abstract

**Objectives:**

The relationship between pain and hypertension is of great pathophysiological and clinical interest in the pain field, but the mechanism is poorly understood. This study used the postoperative patient-controlled analgesia (PCA) dose and the visual analysis scale (VAS) score to assess the relationship between pain and hypertension.

**Methods:**

In this prospective study in a single-center hospital, 200 participants were enrolled and divided into three groups: normotensive group, hypertension without treatment group, and hypertension with treatment group. The participants scheduled for elective inhalational general anesthesia were interviewed at hospital admission.

**Results:**

A significant difference was observed in analgesic dosage on postoperative days 1, 2, and 3 between the female normotensive group and female hypertension with treatment group (*independent-samples, one-way analysis of covariance, age, and weight as covariates:P*=0.021, 0.014, 0.032). No significant differences in the VAS scores and PCA dosages were observed between the male normotensive group and any one of the male hypertensive groups.

**Conclusion:**

We agree that hypertensive hypoanalgesia exists in some experimental settings. The mechanism linking postoperative pain and hypertension is far more complex than we initially believed. Therefore, more studies are required to investigate the roles that antihypertensive drugs, sex, and psychological stress play. Antihypertensive drugs may play a crucial role in mediating the relationship between pain and hypertension. Psychosocial factors were discussed but were not examined.

## 1. Introduction

Hypertension is a widespread problem around the world. There are few articles discussing about the relationship between hypertension and postoperative acute pain. A study on the relationship between hypertension and postoperative pain may offer clinical physicians guidance for pain management in patients with hypertension. Hypertension has long been associated with hypoalgesia [1]. Our previous study revealed the role of a *β*-blocker in anesthesia and postoperative pain management after hysterectomy [2]. Patients in an esmolol group used a lower quantity of PCA morphine than those in a control group in the first 72 postoperative hours. Most studies discussing about relationship between postoperative pain and hypertension have not considered the interference of antihypertensive treatment. Therefore, we designed a prospective observational study comprising three groups (control, hypertension without treatment, and hypertension with treatment) in a single-center hospital to assess the relationship between hypertension and postoperative pain.

## 2. Materials and Methods

The study protocol was approved by the hospital's Institutional Review Board, and all subjects provided informed consent prior to participation. This manuscript adheres to the applicable guidelines in the Consolidated Standards of Reporting Trials.

### 2.1. Design/Subjects

A prospective study was conducted in which potential subjects were identified through systematic review of daily admission logs. Patients (aged 20 to 75 years) scheduled for elective general anesthesia and who were willing to use morphine in postoperative pain control were interviewed at hospital admission. The sample comprised 200 participants who met the inclusion criteria.

#### 2.1.1. Exclusion Criteria

Patients were excluded if they had had liver disease, renal disease, dementia, use of chronic pain medications, conscious disturbance, secondary hypertension and myocardial infarction, cardiac bypass surgery, unstable angina, cerebrovascular events, congestive heart failure, or significant arrhythmia events in the preceding 6 months. Pregnant or breastfeeding patients were also excluded.

Enrolled patients were divided into three groups as follows:

1. Normotensive group (control group)
2. Hypertension without treatment group
3. Hypertension with treatment group, described as follows (according to the patient's daily routine treatment)

### 2.2. Assessment of Essential Hypertension History

Eligible subjects were informed that they were participating in a study on essential hypertension and acute pain management. The normotensive group was defined as not having a systolic pressure over 140 mmHg or diastolic pressure over 90 mmHg more than twice between the admission and interview. Patients who revealed that they had a history of hypertension were enrolled in the essential hypertension group. Patients who did not have a history of hypertension but who had a systolic pressure of over 140 mmHg or a diastolic pressure of over 90 mmHg more than twice between admission and the interview were also placed in the essential hypertension group. Patients with hypertension were divided into two groups, a treatment group and a nontreatment group, according to the use of antihypertensive agents. The treatment and choice of drugs for hypertension were oriented to international guidelines by each patient's clinician. The treatment was divided into four categories: an angiotensin-converting-enzyme inhibitor (ACEI), a *β*-blocker, a calcium channel blocker, and a diuretic.

### 2.3. Induction of Anesthesia and Postoperative Pain Therapy

General inhalation anesthesia was used and maintained using a standardized procedure by anesthesiologists. No premedication was administered to any of the patients. Before the surgery, we taught the patients how to use the PCA system and how to evaluate their own pain intensity by using the visual analogue scale (0: no pain; 10: the worst pain intensity). The PCA system had the following initial settings: a bolus of 1 mg of morphine at the patient's demand, 5 minutes of lockout time, and an upper limit of 20 mg of morphine. The PCA dosage setting was adjusted daily depending on the patient's pain intensity at rest. A trained research assistant obtained information on postoperative pain intensity, PCA consumption, and related side effects from the medical records.

### 2.4. Measurement of Confounders

Information regarding demographic, physiologic, anesthetic, and surgical confounders was obtained using standardized questionnaires, the patients' medical records, and physical examinations. The heights and weights of the participants were obtained from patient intake sheets. The surgical sites were extracted from the operative reports approximately 1 week after admission and were classified as one of the following: (1) upper abdominal, (2) lower abdominal, (3) urologic, (4) orthopedic, and (5) others. Type and duration of anesthesia were extracted from the anesthetic record. Information regarding each patient's essential hypertension history, sex, age, presurgical weight, medical history, and postoperative course were collected. Furthermore, all nonopiate and opiate analgesic use in the initial 72 postoperative hours was recorded.

### 2.5. Statistical Analysis

Independent-samples, one-way analysis of variance (ANOVA) and Tukey's post hoc multiple comparisons were used to test for significant differences among the three groups for continuous variables such as age and body mass index (BMI) (Table 1). The categorical variables, sex and American Society of Anesthesiologists Classification (ASA Class), among the groups were determined using frequency distributions, and chi-square tests were performed to determine whether the distributions differed significantly among the groups. Independent-samples, one-way analysis of covariance (ANCOVA) was used to evaluate the relationship between hypertension or its treatments and postoperative PCA consumption and pain scores by sex, after adjusting for potential confounders. The covariates in independent-samples, one-way ANCOVAs differed significantly among the groups; the covariates were taken from those variables that were determined to be predictors as reported in the literature. Analyses were performed using SPSS (v. 22; IBM Corporation, Armonk, NY, USA). *P* < 0.05 was considered statistically significant.

## 3. Results

A total of 200 participants were recruited until December 2015. The first group of patients (patients without hypertension) comprised 52 subjects; the second group (patients with hypertension without treatment) comprised 82 subjects; and the third group (patients with hypertension under medical treatment) comprised 66 subjects. The demographics of the patients are presented in Table 1. The data were separated by sex due to the differences in presentation in women and men (Tables 2 and 3, respectively). With respect to each sex, we adjusted for age and body weight through independent-samples, one-way ANCOVA to compare the VAS scores and morphine dosages among the three groups. Significant differences were observed in analgesics dosage on postoperative days 1, 2, and 3 in the three female groups (*P*=0.021, 0.014, 0.032). A post hoc test revealed a significant difference between the normotensive group and the hypertension under medication group. Significant differences were also observed in the postoperative pain scores of the male patients in terms of the analogue scales on movement (VASM) on postoperative day 1, the analogue scales at rest (VASR) on postoperative day 1, and the VASM on postoperative day 2 among the three groups (*P*=0.015, 0.038, 0.032). The results of the post hoc test revealed no significant difference between the normotensive group and either of the hypertensive groups; however, a significant difference was observed in the VASM and VASR on postoperative day 1 between the hypertension with and without treatment groups.

## 4. Discussion

The relationship between hypertension and postoperative pain is of great pathophysiological and clinical interest in the pain field, but the mechanism is poorly understood [3]. Hypertensive hypoanalgesia can be observed obviously in an experimental setting [4]. It is a biological defense mechanism that protects individuals from further harm through a baroreceptor that regulates the descending inhibitory pathways of pain that induce endogenous opioids [3]. Most animal and human studies have determined that hypertension is related to hypoanalgesia [5–7]. A study reported an inverse relationship between resting blood pressure and acute pain sensitivity in healthy normotensives [8]. In an animal model, a study showed that the expression of gamma-aminobutyric acid receptors in nociceptive spinal neurons decreases during hypertension; another study showed caudal ventrolateral medullary reticular formation in nociceptive-cardiovascular integration [9, 10]. However, this mechanism is still debated because studies that have used antiopioids have been unable to identify the cause of hypertension-related hypoanalgesia [11–13]. Ring et al. used a double-blinded placebo control design and found that the differences in pain reporting between hypertension and normotensive groups were unaffected by opioid blockade with naltrexone. Their results are congruent with those in most opioid blockade studies [5] that have failed to support the hypothesis that hypertensive hypoalgesia is mediated by endogenous opioids. More research is required to clarify the mechanism of hypertension-related hypoalgesia.

This study determined that female patients with hypertension undergoing treatment required significantly more morphine than did the normotensive patients during the initial 72 hours after operation. No differences in morphine consumption and pain scores were observed between the normotensive group and either of the male hypertensive groups. An increasing number of studies have used PCA dose to evaluate acute postoperative pain [14]. France and Katz observed this relationship by using a patient-controlled analgesia (PCA) pump system in response to an invasive procedure in prostate surgery [4]. They reported that patients with hypertension had lower pain scores than normotensive male patients who underwent radical prostatectomy. However, no differences were observed in PCA dosages, and they could not clearly explain the phenomenon [15]. In their discussion, they suggested that morphine administration suppresses the release of endogenous opiates previously activated in hypertension patients and that individuals with high blood pressure require relatively high PCA dosages. This suggestion is in agreement with our findings in which the female patients with hypertension used more PCA than the normotensive patients. In the same time, the hypertension without medication group used more PCA dosages than the normotensive group, but the difference was nonsignificant. Most studies have not included other factors that confound the relationship between postoperative pain and hypertension. Our study introduced numerous covariates that may have interfered with the results, including sex, hypertension treatment, age, weight, and surgical duration. The results did not reveal direct evidence of the mechanism between postoperative pain and hypertension, but we address important key points that worth discussion. Human hypertension is complex and multifactorial, and distinguishing endocrine neurological components from genetic components is particularly difficult [16]. Therefore, the incidence of hypoalgesia in individuals with a family history of hypertension is epidemiologic, but no causal evidence has been presented [16]. Most data are from Western studies and cannot be correlated with the hypoalgesia and hypertension data in routine use in clinical settings. Our data were derived from only one Asian country (Taiwan), and after controlling for many covariates, we realized that this topic is much more complex than we initially believed.

After comparing the differences in postoperative pain and sex reported in numerous studies, Zheng et al. found that age and preoperative pain were major confounders for sex-based differences in postoperative pain outcomes in a prospective database analysis. We therefore separated the data by sex for analysis. Zheng et al. confirmed that female sex is a significant predictor for postoperative pain after surgery, especially older women (aged over 50 years). We assumed that because the mean age of the female group (mean age: 55.5 years) was older and some of the women may have been menopausal, more complex hormonal effects would have been present than in the male group [17]. Because the female and male patients had contrary presentations in our study, we observed no hypertensive hypoanalgesia effect in the male patients but observed a “reverse” hypertensive hypoanalgesia effect in the female patients [18]. This finding is in agreement with the findings of a 1999 study, in which sex-based differences in pain responses to the cold pressor test in individuals with a positive or negative parental history of hypertension were investigated [19, 20]. The findings suggested that the generalizability of the hypoalgesic effects in women prone to hypertension should be investigated. On the other hand, hypertensive female with antihypertensive drugs use more morphine dosages than normotensive female. The antihypertensive drugs however impacted the results among the female patients through an unknown mechanism; moreover, the male patients with hypertension who did not use antihypertensive drugs reported more pain on postoperative day 1 than did those who use antihypertensive drugs. In an animal study, mice treated orally with several ACEIs or AT1RAs developed hypoalgesia; [21] however, more studies are required, particularly with antihypertensive drugs, to understand the mechanism [22]. Palhares et al. reported a synergistic antinociceptive effect from a calcium channel blocker and a TRPV1 blocker in an acute pain model in mice [23]. In a human study, the mechanisms acting through both antihypertensive and specific pharmacodynamic properties may account for the normalization of pain sensitivity observed in patients with hypertension during ACEI treatment. Physicians currently prescribe antihypertensive drugs by using several core principles for treating hypertension, including a low dosage and combined therapy [24]. These core principles make it difficult for studies like ours to define which categories of antihypertensive drugs help patients control their hypertension. We defined four categories of antihypertensive treatment at the beginning of this study, namely an ACEI, a *β*-blocker, a calcium channel blocker, and a diuretic. We did not have sufficient case numbers to analyze the subgroup in the patients undergoing antihypertension treatment because most of our patients received multiple descriptions; future studies are required to confirm the findings of this study.

As already stated, morphine administration may suppress endogenous opiate release. We attempted to determine why women with hypertension use a larger morphine dose than normotensive women. From a psychosocial aspect, pain not only involves a central inhibitory mechanism but also an emotional element. In a review article [25], the authors examined the relationship between psychosocial risk factors and hypertension. Sixteen of the studies reported an association between psychosocial stressors and blood pressure and demonstrated how the association plays a vital role in the development of hypertension. One 1993 study [26] reported that preoperative and postoperative emotional distress factors were significantly related to the dose/demand ratio and hourly analgesic usage. Although we did not assess the psychosocial stress in our patients, according to the abovementioned hypothesis, female patients with hypertension have more psychosocial stress than normotensive female patients and have greater postoperative morphine requirements. This is especially true for patients treated using antihypertensive drugs. Female patients with hypertension may require more clinical observation during postoperative care, which can be provided through multimodal pain treatment, including perioperative psychological support [27].

### 4.1. Study Limitations

1. We did not assess psychosocial stress in our patients before and after the surgery
2. We did not determine whether the patients (in the hypertension with treatment group) controlled their hypertension properly using the prescriptions
3. This study did not conduct a double-blinded experiment; therefore, the patients might have known they were in dosage research and cause participant bias
4. We did not have enough case numbers to assess the role of each type of antihypertensive drug

## Figures and Tables

**Table 1:** Demographic data for the three groups (*N*=200).

	Normotensive (*N*=52)	H/T without M (*N*=82)	H/T with M (*N*=66)	*P* value
Age (y/o)	49.2 ± 11.7^†¶^	56.8 ± 10.2^‡^	61.2 ± 8.6	<0.001^*∗*^
SBP	119.4 ± 16.8^†¶^	143.6 ± 21.1	144.6 ± 22.1	<0.001^*∗*^
DBP	65.1 ± 13.7^†¶^	78.0 ± 13.4	75.36 ± 15.9	<0.001^*∗*^
Sex (male/female)	12/40	39/43	28/38	0.015^*∗*^
Height (cm)	159.1 ± 8.0	160.6 ± 8.8	158.9 ± 7.6	0.378
Weight (kg)	59.9 ± 12.1^†¶^	66.2 ± 12.8	66.1 ± 10.8	0.006^*∗*^
ASA (I/II/III)	12/35/5	5/63/14	0/48/18	<0.001^*∗*^
BMI	23.6 ± 3.7^†¶^	25.6 ± 4.2	26.2 ± 4.4	0.002^*∗*^
Surgical time (hr)	5.4 ± 2.9	5.1 ± 2.2	5.6 ± 2.5	0.443
Intraoperative fluid infusion (mL)	1107.9 ± 1173.4	1252.5 ± 1363.2	1455.2 ± 1367.3	0.354
Intraoperative blood loss (mL)	1576.6 ± 1912.9	1206.6 ± 1309.5	1375.3 ± 1601.8	0.418
Intraoperative urine output (mL)	449.0 ± 488.6	366.4 ± 388.7	545.6 ± 663.9	0.124

Data presented as mean ± standard deviation (SD) or number (%). SBP: systolic blood pressure; DBP: diastolic blood pressure; BMI: body mass index; H/T without M: hypertension without medication. ^*∗*^ *P* < 0.05 when using independent-samples, one-way ANOVA. ^¶^ *P* < 0.05 when comparing the normotension and H/T without medication groups. ^‡^ *P* < 0.05 when comparing the H/T without medication and H/T with medication groups. ^†^ *P* < 0.05 when comparing the normotension and H/T with medication groups.

**Table 2:** Comparisons of postoperative outcomes in female patients unadjusted and adjusted for age and weight (*N*=121).

		Normotensive (*N*=40)	H/T without M (*N*=43)	H/T with M (*N*=38)	*P* value	Adjusted *P* value
POD1	VASM1	4.3 ± 1.9	4.6 ± 1.9	4.6 ± 1.8	0.662	0.909
	VASR1	1.9 ± 1.6	2.4 ± 1.7	2.1 ± 1.8	0.364	0.305
	DOSE1 (mL)	61.0 ± 34.4	68.6 ± 39.7	74.4 ± 44.9^†^	0.330	0.021^!^
	PONV1	1 (2.5%)	2 (4.6%)	4 (10.5%)		

POD2	VASM2	3.3 ± 1.8	3.6 ± 1.3	3.6 ± 1.6	0.604	0.771
	VASR2	1.2 ± 1.5	1.5 ± 1.3	1.3 ± 1.1	0.593	0.588
	DOSE2 (mL)	101.7 ± 49.0	115.5 ± 66.4	125.1 ± 56.7^†^	0.206	0.014^!^
	PONV2	1 (2.5%)	2 (4.6%)	0 (0%)		

POD3	VASM3	2.5 ± 1.6	2.8 ± 1.7	2.7 ± 1.3	0.734	0.750
	VASR3	0.9 ± 0.9	0.9 ± 1.0	0.7 ± 0.9	0.710	0.808
	DOSE3 (mL)	132.4 ± 71.4	144.4 ± 91.1	157.9 ± 71.4^†^	0.366	0.032^!^
	PONV3	0 (0%)	0 (0%)	1 (2.6%)		

Data are presented as mean ± SD or number (%). POD1: postoperative day 1; POD2: postoperative day 2; POD3: postoperative day 3; PONV: postoperative nausea and vomiting; VASM: analogue scales on movement; VASR: analogue scales at rest; DOSE: postoperative morphine consumption (0.4 mg/mL). ^!^Adjusted *P* < 0.05 for independent-samples, one-way ANCOVA (age and weight as covariates). ^+^Adjusted *P* < 0.05 when comparing the normotensive and H/T with medication groups.

**Table 3:** Comparisons of postoperative outcomes in male patients unadjusted and adjusted for age and weight (*N*=79).

		Normotensive (*N*=12)	H/T without M (*N*=39)	H/T with M (*N*=28)	*P* value	Adjusted *P* value
POD1	VASM1	4.3 ± 1.1	5.0 ± 1.9^‡^	3.7 ± 1.3	0.007^*∗*^	0.015^!^
	VASR1	1.8 ± 1.1	2.3 ± 1.7^‡^	1.3 ± 1.1	0.042^*∗*^	0.038^!^
	DOSE1 (mL)	86.7 ± 62.0	77.9 ± 41.3	72.9 ± 35.9	0.651	0.919
	PONV1	0 (0%)	1 (2.5%)	2 (7.1%)		

POD2	VASM2	3.3 ± 1.0	3.9 ± 1.6	3.2 ± 1.5	0.133	0.032!
	VASR2	1.2 ± 0.8	1.3 ± 1.2	0.9 ± 1.1	0.401	0.362
	DOSE2 (mL)	114.5 ± 84.0	133.7 ± 62.8	135.9 ± 66.0	0.888	0.761
	PONV2	0 (0%)	0 (0%)	0 (0%)		

POD3	VASM3	2.2 ± 1.3	3.1 ± 1.7	2.5 ± 1.1	0.273	0.085
	VASR3	0.6 ± 0.6	0.7 ± 0.8	0.5 ± 0.5	0.476	0.578
	DOSE3 (mL)	171.0 ± 101.6	164.7 ± 83.1	169.6 ± 82.2	0.961	0.564
	PONV3	0 (0%)	0 (0%)	0 (0%)		

Data are presented as mean ± SD or number (%). POD1: postoperative day 1; POD2: postoperative day 2; POD3: postoperative day 3; PONV: postoperative nausea and vomiting; VASM: analogue scales on movement; VASR: analogue scales at rest; DOSE: postoperative morphine consumption (0.4 mg/mL). ^*∗*^Unadjusted *P* < 0.05 for independent-samples, one-way ANOVA. ^!^Adjusted *P* < 0.05 for independent-samples, one-way ANCOVA (age and weight as covariates). ^‡^Adjusted *P* < 0.05 when comparing the H/T without medication and H/T with medication groups.

## Data Availability

The data used to support the findings of this study are included within the supplementary information file.

## Supplementary Materials

(Supplementary Materials) Hypertension_pain.csv is our raw data that can be assessed for public.
